# Genotypic and phenotypic diversity of *Bacillus *spp. isolated from steel plant waste

**DOI:** 10.1186/1756-0500-1-92

**Published:** 2008-10-17

**Authors:** Dulcecleide B Freitas, Mariana P Reis, Cláudia I Lima-Bittencourt, Patrícia S Costa, Paulo S Assis, Edmar Chartone-Souza, Andréa MA Nascimento

**Affiliations:** 1 Departamento de Biologia Geral, Instituto de Ciências Biológicas, Universidade Federal de Minas Gerais, Belo Horizonte, Brazil; 2 Departamento de Engenharia Metalúrgica e de Materiais, Escola de Minas, Universidade Federal de Ouro Preto, Ouro Preto, Brazil

## Abstract

**Background:**

Molecular studies of *Bacillus *diversity in various environments have been reported. However, there have been few investigations concerning *Bacillus *in steel plant environments. In this study, genotypic and phenotypic diversity and phylogenetic relationships among 40 bacterial isolates recovered from steel plant waste were investigated using classical and molecular methods.

**Results:**

16S rDNA partial sequencing assigned all the isolates to the *Bacillus *genus, with close genetic relatedness to the *Bacillus subtilis *and *Bacillus cereus *groups, and to the species *Bacillus sphaericus*. tDNA-intergenic spacer length polymorphisms and the 16S–23S intergenic transcribed spacer region failed to identify the isolates at the species level. Genomic diversity was investigated by molecular typing with rep (repetitive sequence) based PCR using the primer sets ERIC2 (enterobacterial repetitive intergenic consensus), (GTG)_5_, and BOXAIR. Genotypic fingerprinting of the isolates reflected high intraspecies and interspecies diversity. Clustering of the isolates using ERIC-PCR fingerprinting was similar to that obtained from the 16S rRNA gene phylogenetic tree, indicating the potential of the former technique as a simple and useful tool for examining relationships among unknown *Bacillus *spp. Physiological, biochemical and heavy metal susceptibility profiles also indicated considerable phenotypic diversity. Among the heavy metal compounds tested Zn, Pb and Cu were least toxic to the bacterial isolates, whereas Ag inhibited all isolates at 0.001 mM.

**Conclusion:**

Isolates with identical 16S rRNA gene sequences had different genomic fingerprints and differed considerably in their physiological capabilities, so the high levels of phenotypic diversity found in this study are likely to have ecological relevance.

## Background

The genus *Bacillus *encompasses Gram-positive rod-shaped, endospore-forming aerobic or facultatively anaerobic bacteria, and is a phenotypically and phylogenetically diverse taxon [1]. *Bacillus *species are distributed widely in nature. *Bacillus cereus *and *Bacillus anthracis*, both of which belong to the *B. cereus *group [2], are amongst those associated with human disease. Using 16S rDNA sequence analysis, Ash et al. [3] described the presence of five phylogenetically distinct groups in the genus *Bacillus*, and Nielsen et al. [4] subsequently described a sixth group belonging to the alkaliphilic bacilli. Two of these groups are the *B. cereus *group (*B. anthracis, B. cereus, Bacillus thuringiensis, Bacillus mycoides, Bacillus pseudomycoides and Bacillus weihenstephanensis*) and the *Bacillus subtilis *group (*B. subtilis, Bacillus pumilus, Bacillus atrophaeus, Bacillus licheniformis and Bacillus amyloliquefaciens*) [3].

Since the genus *Bacillus *includes species of industrial, biotechnological and environmental interest, as well as clinically important species, various studies have been made of its genetic diversity. However, it is still difficult to identify and characterize new isolates, mainly because some species share morphological and biochemical characteristics [3,5,6], which makes it hard to separate them. In addition, species isolated from the environment have considerable diversity with respect to physiology, DNA G + C content and nutritional requirements [1,3,6,7]. Molecular approaches are increasingly being used for rapid species identification [8]. Various techniques, including tDNA-PCR (tDNA-intergenic spacer length polymorphisms) and ITS-PCR (16S–23S intergenic transcribed spacer region) analyses have been used for identification and differentiation of bacterial species [9,10]. Repetitive element sequence-based PCR (rep-PCR) genomic fingerprinting has also been used for discriminating among a wide range of bacterial genera and species, and to compare bacterial genome diversity. This method can be used to generate more accurate information because it is capable of screening several parts of the bacterial genome [11,12].

Many studies have investigated *Bacillus *isolates originating from hospital, food and environmental samples [13], but little attention has been given to isolates recovered from steel-making wastes [14]. Blast furnace sludge (BFS) contains tramp elements such as Zn, Ca, Mg and Al, preventing its recycling for steel production or other purposes. As steel industries generate about 700 kg of waste per ton of steel produced, the removal or reduction of the tramp elements by microorganisms could make steel-waste recycling viable, and provide ecological and economic benefits.

As the microbial communities in steel-making wastes have rarely been examined, the purpose of this study was to investigate the genotypic and phenotypic diversity of 40 *Bacillus *isolates obtained from this environment. This was based on biochemical (API 50 CHB/E), physiological (including susceptibility to heavy metals) and molecular approaches. 16S rRNA gene sequencing, tDNA-PCR and ITS-PCR analyses were used for taxonomic identification of the isolates and to reveal phylogenetic relationships among them. Other methods used in genetic characterization and differentiation of the isolates included ERIC, BOX and (GTG)_5_.

## Methods

### Sampling and bacterial isolates

Blast furnace sludge (BFS) was sampled in triplicate from a steel plant (Acesita-Cia Aços Especiais, Minas Gerais state, Brazil) using sterilized bottles. BFS is produced after the first step in ore processing, when the raw material is subjected to high temperatures for ore separation. The chemical composition of BFS was determined by Mössbauer and X-ray analyses as: 38.72% FeT, 6.74% Si, 4.60% Zn, 2.29% Ca, 2.14% Mg, 1.81% Al, and traces of Cr, S, P and Mn (< 1%).

Isolates were recovered from the BFS by blending 1 g wet weight of the sample with 9 ml phosphate buffer and shaking at 37°C for 24 h before serial dilution and plating onto 1/10 strength tryptic soy agar (Difco). The resulting colonies were purified by restreaking on the same medium and incubating at 37°C, prior to use in molecular and phenotypic analyses. The type strains of *B. subtilis *(ATCC 6633T), *B. licheniformis *(ATCC 14580T), *B. pumilus *(ATCC 7061T), *B. amyloliquefaciens *(ATCC 23842), *B. cereus* (ATCC 11778) and *B. sphaericus *(ATCC 14577T) were included as reference strains.

### Morphological, physiological and biochemical characteristics

Morphological and physiological characterization of the 40 isolates was based on the Gram reaction, shape, motility, endospore formation, and growth at different temperatures and NaCl concentrations. To confirm molecular taxonomic identification of isolates as *B. subtilis *and *B. pumilus*, the ability to hydrolyze starch was investigated. The isolates were also identified according to their biochemical profiles using the API 50CH/B test kit (BioMérieux, Marcy l'Etoile, France), following the manufacturer's instructions, and according to their utilization of citrate and indol, and hydrogen sulphide production, performed as described previously [15]. API test kit results were interpreted using the Analytical Profile Index (API) database of the Apiweb software (version 4.0; BioMérieux, Marcy l'Etoile, France).

The minimum inhibitory concentrations (MIC) of heavy metals (mercury chloride, silver nitrate, lead nitrate, cobalt chloride, copper sulphate, zinc sulphate and nickel chloride) were determined by a serial two-fold agar dilution method using nutrient agar (Difco). All heavy metals were purchased from Sigma-Aldrich (St. Louis, MO, USA).

### DNA extraction, PCR amplification, sequencing of 16S rDNA, and phylogenetic analysis

Genomic DNA of the isolates was prepared as described previously [16]. 16S rRNA genes were amplified using the primers 8F and 907R, and the amplification cycling conditions [see Additional file 1]. All sequencing reactions were performed using the Dynamic ET Dye (Amersham BioSciences) sequencing kit, and reactions were analyzed on a MegaBACE 1000 capillary sequencer (Amersham BioSciences). Sequences were compared with available databases using the GenBank BLASTN and RDP Classifier search tools to determine approximate phylogenetic affiliations. To accomplish this, the partial 16S rRNA gene sequences were basecalled, checked for quality, aligned and analyzed using Phred v.0.20425 [22], Phrap v.0.990319 [23] and Consed 12.0 [24] software. The phylogenetic relationships were inferred by MEGA 3.1 [25] using the Neighbor-Joining (N-J) method and the Kimura 2-P model of sequence evolution. The robustness of the phylogenetic tree topology was evaluated with 1000 replicates of bootstrap analysis [26,27]. The nucleotide sequences generated were deposited in the GenBank database with accession numbers EU689117 to EU689157.

### tDNA-PCR, ITS-PCR and rep-PCR DNA fingerprinting

Three variations of rep-PCR genomic fingerprinting were performed using the ERIC, BOX and (GTG)5 primers. The primers and the amplification cycling conditions for the rep-PCR, tDNA and ITS-PCR [see Additional file 1]. Products were separated by electrophoresis in 2% agarose in 1 X TBE buffer for 3.5 h at 65V, and visualized by staining with ethidium bromide (0.5 mg/mL). Fingerprints generated were compared visually. The reproducibility of the fingerprint profiles obtained was assessed in at least three separate experiments.

### Cluster analysis

For cluster analysis, the data were converted to a binary matrix, where the digits 1/0 represent the presence/absence of a phenotypic character or DNA band. The similarity matrix was generated by Euclidean distances, which were used to build a tree with the unweighted pair group mean averages (UPGMA) algorithm. Analysis of data was performed using the software PAST [28].

## Results

**Figure 1:**
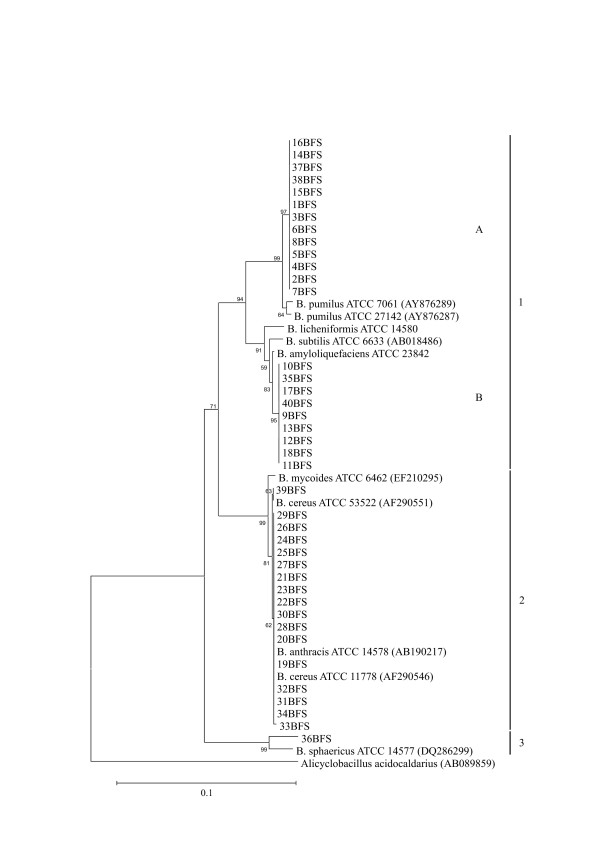
Phylogenetic tree of members of the genus *Bacillus*, based on 16S rRNA gene sequences. The tree was constructed using the neighbor-joining method, and genetic distances were computed by using Kimura's model. Numbers at nodes indicate percentages of occurrence in 1000 bootstrapped trees. *Alicyclobacillus acidocaldarius *(AB089859) was used as an outgroup.

The 16S rDNA sequences used for phylogenetic analyses were 666 nucleotides long and spanned the V2 to V5 variable regions, corresponding to *Escherichia coli *K12 16S rDNA. The 16S rDNA sequences of the reference strains used in the study were obtained from GenBank, except for those of *B. amyloliquefaciens *ATCC 23842. The phylogenetic tree based on these sequences revealed close relationships among the isolates and with other members of the genus *Bacillus *(Fig. 1).

Based on 16S rDNA sequence analysis, 13 isolates clustered with the *B. pumilus *strains ATCC 7061 and ATCC 27142 at 99.8% similarity. Nine isolates had a sequence similarity of 99.9% with *B. amyloliquefaciens *(ATCC 23842). Slightly lower similarities (99.7% and 99.5%) were found for these nine isolates with *B. subtilis *(ATCC 6633) and *B. licheniformis *(ATCC 14580), respectively. Seventeen isolates clustered at 99.9% sequence similarity with the *B. cereus *group (*B. cereus, B. mycoides *and *B. anthracis*). Fifteen of these 17 isolates had identical 16S rRNA gene sequences. The sequence of isolate 36BFS had 97.1% similarity with *B. sphaericus* (ATCC 14577).

**Figure 2:**
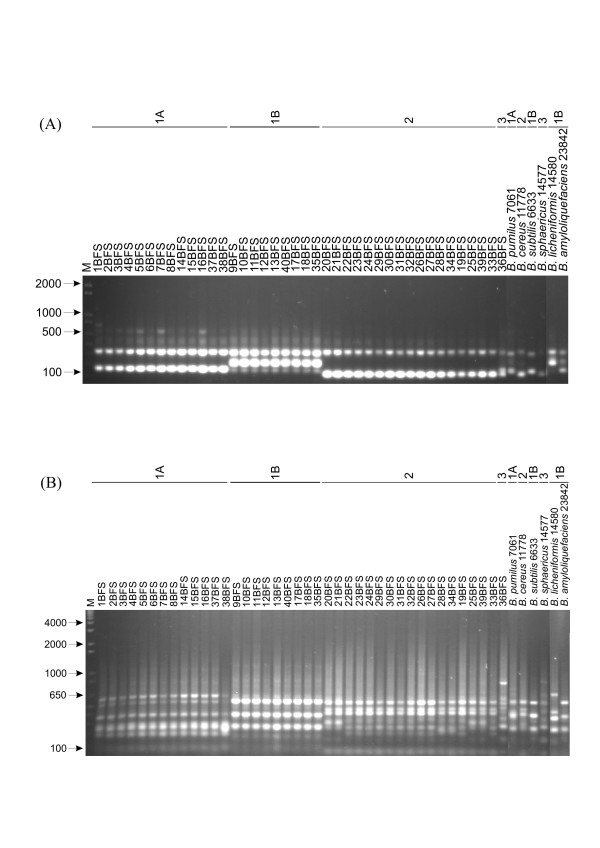
(A) ITS- and (B) tDNA-PCR fingerprinting of isolates and reference strains. Lane M, molecular size marker (1 Kb Plus-Invitrogen). The numbers above the figure identify the 16S rDNA-sequence-based phylogeny clusters obtained for the isolates.

ITS and tDNA-PCR are used as molecular markers to differentiate isolates at the species level. The resolution powers of ITS-PCR and tDNA-PCR were evaluated using the reference strains of *Bacillus *species identified from the 16S rRNA gene sequencing analyses (Fig. 1). The ITS and tDNA-PCR fingerprinting gave 10 and 11 distinct patterns, respectively (Fig. 2A and 2B). The *Bacillus *isolates, grouped on the basis of 16S rRNA gene sequence phylogeny, had identical ITS and tDNA-PCR patterns, except for the tDNA-PCR of four isolates of the *B. cereus *group. These patterns differed from those of all the reference strains.

**Figure 3:**
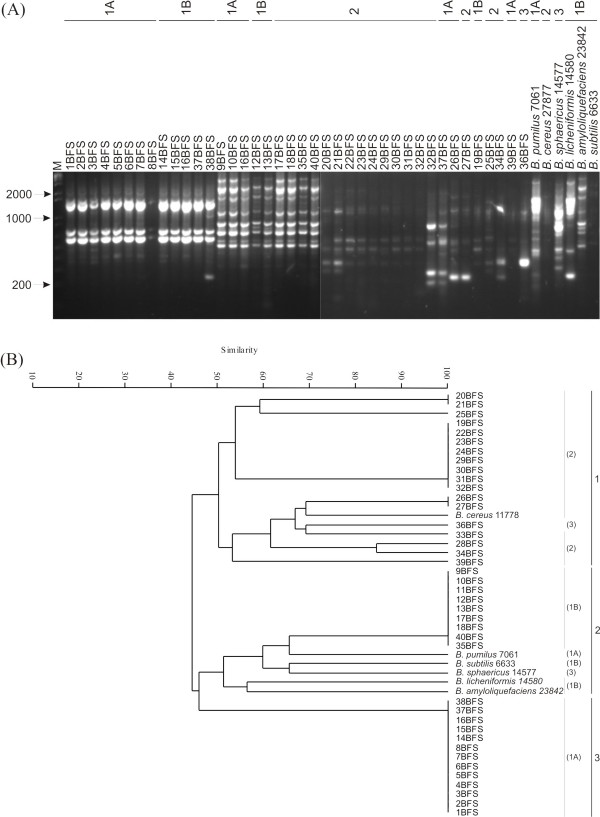
(A) ERIC-PCR fingerprinting patterns of the isolates and reference strains. Lane M, molecular size marker (1 Kb plus-Invitrogen). The numbers above the figure identify the 16S rDNA-sequence-based phylogeny clusters obtained for the isolates. (B) UPGMA cluster analysis of isolates and reference strains based on ERIC-PCR. Numbers in parentheses identify the 16S rDNA-sequence-based phylogeny clusters obtained for the isolates.

**Figure 4:**
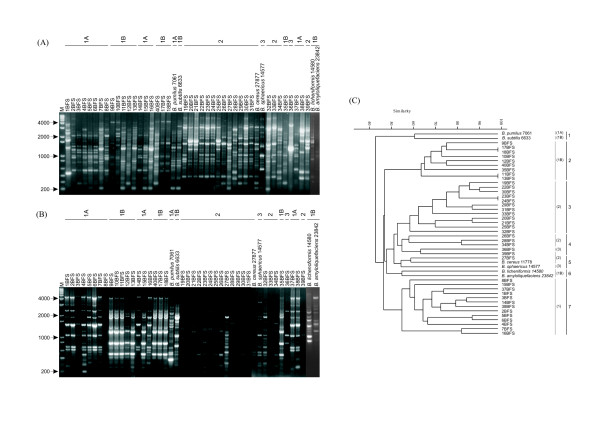
(A) (GTG)_5_-PCR, (B) BOX-PCR fingerprinting patterns of the isolates and reference strains. Lane M, molecular size marker (1 Kb Plus-Invitrogen). The numbers above the figure identify the 16S rDNA-sequence-based phylogeny clusters obtained for the isolates. (C) UPGMA cluster analysis of isolates and reference strains based on multi rep-PCR global matrix of ERIC-PCR, BOX-PCR and (GTG)5-PCR. Numbers in parentheses identify the 16S rDNA-sequence-based phylogeny clusters obtained for the isolates.

Rep-PCR genomic fingerprinting has been used in many organisms to infer phylogenetic relationships among isolates, and to study their diversity in a variety of ecosystems. The fingerprint patterns of the 40 isolates generated by ERIC, (GTG)_5 _and BOX-PCR were complex, producing a large number of polymorphic bands of variable intensity (Figs 3A, 4A and 4B). Thirty-six bands were identified by BOX-PCR (approximately 200 to 4000 bp), 34 bands by ERIC-PCR (200 to 4000 bp) and 49 bands by (GTG)_5_-PCR (200 to 4000 bp). All reference strains had unique profiles in all genomic fingerprinting analyses, and did not match any of the patterns obtained for the *Bacillus *isolates. Among the three sets of primers, (GTG)_5_-PCR fingerprinting gave the broadest band pattern. BOX-PCR amplifications were negative for two isolates (8BFS and 31BFS), based on three independent PCRs. Negative results were also observed with ERIC-PCR fingerprinting for isolate 33BFS. The dendrogram based on ERIC-PCR fingerprinting analysis (Fig. 3B) showed a clustering of isolates similar to that of the 16S rDNA phylogenetic tree. With ERIC-PCR, only isolate 36BFS (cluster 1) grouped outside its 16S rDNA phylogenetic tree cluster (cluster 3, Fig. 1). The dendrogram generated from multi-rep-PCR fingerprinting (Fig. 4C) revealed considerable genetic heterogeneity among the isolates and reference strains.

The API 50 CH system was used to assist typing of the isolates to the species level, and to study their phenotypic diversity. Data from API 50CH/B and additional physiological tests [see Additional file 2]. All 40 isolates were motile, Gram-positive, spore-forming rods. Fifteen grew at 55°C and 12 grew at 8°C, while 32 and 13 isolates were able to grow in 7.5% and 15% NaCl, respectively. Only two of the isolates used citrate as a sole carbon source and three isolates produced hydrogen sulphide. The most commonly used substrates were esculin and D-ribose, and the least commonly used were inulin and potassium gluconate. In an attempt to discriminate *B. pumilus *species among the 22 isolates that clustered within the *B. subtilis *group, the ability to hydrolyze starch was tested. Five of the isolates were starch-hydrolysis-negative, placing them close to *B. pumilus*.

**Figure 5:**
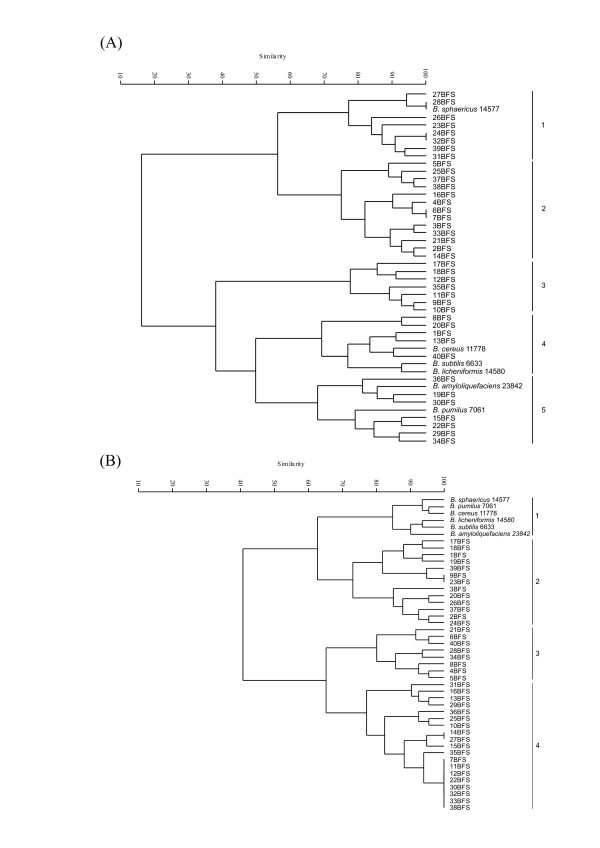
Dendrograms constructed by UPGMA with the *Bacillus *isolates and reference strains according to (A) API 50CH/B profiles and (B) to heavy metal susceptibility profiles.

The 40 isolates were assigned to genus or species based on their API 50CH/B biochemical profiles. Most (n = 28) were assigned to the genus *Bacillus *at a confidence level greater than 90%, while the remaining 12 isolates were identified to the species level as *B. pumilus *(n = 6), *B. cereus *(n = 1), *Bacillus coagulans *(n = 1), *Brevibacillus laterosporus *(n = 1) and *Aneurinibacillus aneurinilyticus *(n = 3). Based on the API identification data, the 40 isolates were highly similar to 3 genera of the Bacillaceae and Paenibacillaceae families. This system failed to identify all of the reference strains to the species level. Moreover, based on the dendrogram generated from the biochemical profile data (Fig. 5A), we observed that most of the isolates used unique combinations of the growth substrates tested.

In the heavy metal assays the highest minimum inhibitory concentrations (MICs) were found for Cu and Pb (4 mM, Table 1). The MIC levels were lower for Zn, Ni, Co and Hg, with 90% of the isolates being inhibited by concentrations ranging from 0.025 to 2 mM. All isolates were inhibited by Ag at the lowest concentration tested. An MIC-based dendrogram revealed profiles of distinct combinations of heavy metals for almost all isolates. The reference strains formed a separate cluster (Fig. 5B). No significant relationship was found between isolate clustering based on this characteristic versus their phylogenetic position.

**Table 1:** Minimum inhibitory concentrations (MICs) of heavy metals for the *Bacillus *isolates from steel plant wastes.

Metal	MIC (mM)		
	
	Range	MIC_50_	MIC_90_
Cu	0.001–4	0.5	4
Pb	0.001–4	2	4
Zn	0.001–4	1	2
Ni	0.001–2	0.25	1
Co	0.001–1	0.25	0.25
Hg	0.001–1	≤ 0.001	0.025
Ag	0.001–1	≤ 0.001	≤ 0.001

Cu, copper sulphate; Pb, lead nitrate; Zn, zinc sulphate; Ni, nickel chloride; Co, cobalt chloride; Hg, mercury bichloride; Ag, silver nitrate.

## Discussion

It is well known that 16S rDNA sequences are good indicators of phylogenetic relationships among bacteria at the intra- and interspecies levels. In our study the 16S rRNA gene sequence analyses yielded very good identification of the isolates at the genus (*Bacillus*) level. As expected, analysis of 16S rRNA gene sequences alone was not sufficient to identify *Bacillus *species, as has also been reported in other studies [29,30]. Identification of the bacteria using the API CHB50 system generally identified isolates to the genus level, and in some cases to the species level, but the identifications obtained using the API system and by 16S rDNA sequencing differed. Nevertheless, biochemical and physiological test data were useful in identifying characteristics that are considered typical of the genus *Bacillus*, allowing phenotypic characterization of the isolates.

Identifications based on 16S rRNA gene sequences are often limited, so fingerprint methods have been developed to characterize and distinguish *Bacillus *isolates [7,9-11]. We used ITS-PCR, tDNA-PCR and rep-PCR genomic fingerprinting to examine phylogenetic relatedness among the isolates. Overall, the isolates phylogenetically closest to *B. sphaericus *and *B. pumilus*, and to the *B. subtilis *and *B. cereus *groups were clearly different from the reference strains.

ITS and tDNA-PCR are frequently used to identify bacterial species and to analyze their phylogenetic relationships [9]. Although there was sufficient resolution to differentiate among reference strains, it was not possible to associate the *Bacillus *isolates with the reference strains using this technique.

Genotype is less affected by environmental factors than phenotype, so the rep-PCR method was used to determine intraspecies diversity among the different *Bacillus *isolates. We found that isolates with the same partial 16S rDNA sequence often had quite dissimilar rep-PCR patterns. The primer specific for (GTG)_5 _was less efficient than the BOX and ERIC primer sets for grouping the isolates. ERIC-PCR fingerprinting and 16S rDNA phylogenetic analyses gave similar clusters. ERIC-PCR fingerprinting clustered reference and environmental *B. cereus *strains in the same way as the 16S rDNA tree, suggesting the former is a good approach to examining genetic relationships among unknown *Bacillus *isolates. The dendrogram generated from multi-rep-PCR fingerprinting separated the environmental *Bacillus *isolates as a distinct group from the reference strains. There was less similarity among the reference strains than among the *Bacillus *isolates, perhaps reflecting their different origins.

Although the isolates had identical 16S rRNA gene sequences, they demonstrated considerable genotypic and phenotypic heterogeneity. Almost all isolates used a distinctive combination of API 50CH/B substrates, suggesting that each occupied a different ecological niche and revealing the co-existence of phylogenetically closely related bacteria in the same environment. Similar results were obtained in relation to heavy metal susceptibility. However, the reference strains clustered apart from the isolates, suggesting that the latter have more similar phenotypes despite their genotypic differences, probably owing to their shared environmental origin; this is contrast to the dendrogram derived from the API 50CH/B profiles.

Although the API 50CH/B system failed to identify the isolates and reference strains to the species level, it was useful for biochemical characterization and revealed significant variability among the isolates. Based on an inability to hydrolyze starch, 5 of the 22 isolates of the *B. subtilis *group were affiliated with *B. pumilus*. The isolates that affiliated with *B. pumilus *by molecular analysis (16S rDNA) had greater physiological diversity.

## Conclusion

Based on molecular characterization, most of the isolates were closely related to the species *B. pumilus *and *B. sphaericus*, and the *B. subtilis *and *B. cereus *groups. Despite the polyphasic approach, it was difficult to identify the environmental *Bacillus *isolates at the species level. We found high genotipic and phenotypic heterogeneity in the *Bacillus *isolates, despite their common origin from a single steel-making waste source.

## Competing interests

The authors declare that they have no competing interests.

## Authors' contributions

DBF carried out the laboratory work and wrote the draft manuscript. MPR and PSC helped DBF with the laboratory work. CIL-B was responsible for computational analysis together with DBF. PSA was responsible for the chemical analysis of the samples from the steel plant waste. ECS contributed to discussion of the results and the draft manuscript. AMAN conceived and designed the study, coordinated the project and helped to write the final manuscript. All authors have read and approved the final manuscript.

## Supplementary Material

Additional file 1 Primer sequences and amplification cycling conditions for the PCR-based genomic DNA fingerprints and 16S rDNA. The primers and the amplification cycling conditions for the rep-PCR, tDNA and ITS-PCR used in this study. Click here for file

Additional file 2 Phenotypic characteristics of the isolates and reference strains used in this study. Data from API 50CH/B and additional physiological tests. Click here for file
