# Supplementary material for: Genotypic and phenotypic diversity of Bacillus spp. isolated from steel plant waste
Source: BMC Res Notes. 2008 Oct 17;1:92. doi: 10.1186/1756-0500-1-92 (PMC2588453; doi:10.1186/1756-0500-1-92)
Supplement: Additional file 1 — Primer sequences and amplification cycling conditions for the PCR-based genomic DNA fingerprints and 16S rDNA. The primers and the amplification cycling conditions for the rep-PCR, tDNA and ITS-PCR used in this study. [file 1756-0500-1-92-S1.doc]

**Primer sequences and amplification cycling conditions for the PCR-based genomic DNA fingerprints and 16S rDNA**.

| Primer set | Primer | Nucleotide sequence 5’- 3’ | References | Primer concentration (µM) | Thermal cycling conditions |
| --- | --- | --- | --- | --- | --- |
| 16S rDNA | 8F | AGAGTTTGATYMTGGCTCAG (271 to 290*) | [19] | 0.5 | Initial denaturation 94°C for 5 min, 94°C for 1 min, 57°C for 1 min and was then decreased by 1°C every two cycles until it was 49°C. Ten additional cycles were carried out at 49°C, 72°C for 3 min, final extension at 10 min at 72°C. |
|  | 907R | CCGTCAATTCMTTTRAGTTT (1356 to 1375*) | [19] |  |  |
|  | PA | TCCTGGCTCAGATTGAACGC (17 to 36*) | [20] |  |  |
|  | U2 | ATCGGYTACCTTGTTACGACTTC (1513 to 1491*) | [21] |  |  |
| tDNA | T5A | AGTCCGGTGCTCTAACCAACTGAG | [22] | 0.5 | Initial denaturation 94°C for 10 min, 30 cycles of 94°C for 30s, 50°C for 30s, 72°C for 1 min, final extension at 72°C for 10 min |
|  | T3B | AGGTCGCGGGTTCGAATCC | [22] |  |  |
| ITS | L1 | CAAGGCATCCACCGT | [23] |  |  |
|  | G1 | GAAGTCGTAACAAGG | [23] |  |  |
| ERIC | ERIC2 | AAGTAAGTGACTGGGGTGAGCG | [11] | 0.5 | Initial denaturation 94°C for 5 min, 30 cycles of 94°C for 1 min, 52°C for 1 min, 72°C for 3 min, final extension at 72°C for 10 min |
| BOX | BOXA1R | CATACGGCAAGGCGACGCT | [11] | 0.4 |  |
| GTG | GTG_5_ | GTGGTGGTGGTGGTG | [11] | 0.3 |  |

**E. coli* K12 16S rRNA gene
