# Supplementary material for: Genotypic and phenotypic diversity of Bacillus spp. isolated from steel plant waste
Source: BMC Res Notes. 2008 Oct 17;1:92. doi: 10.1186/1756-0500-1-92 (PMC2588453; doi:10.1186/1756-0500-1-92)
Supplement: Additional file 2 — Phenotypic characteristics of the isolates and reference strains used in this study. Data from API 50CH/B and additional physiological tests. [file 1756-0500-1-92-S2.doc]

**Phenotypic characteristics of the isolates and reference strains used in this study.**

| Biochemical characteristic | Percentage of positive isolates | | | | | | | | |
| --- | --- | --- | --- | --- | --- | --- | --- | --- | --- |
|  | *B. pumilus* (n=5)* | *B. pumilus* ATCC 7061 | *B. subtilis* group (n=17) | *B. subtilis* ATCC 6633 | *B. amyloliquefaciens* ATCC 23842 | *B. licheniformis* ATCC 14580 | *B. cereus* group (n=17) | *B. cereus* ATCC 6633 | *B. sphaericus* (n=1) |
| Growth at 55 ºC | 60 | - | 47 | - | - | + | 24 | - | - |
| Growth at 8 ºC | 20 | - | 12 | - | + | + | 53 | - | - |
| Growth in 7,5% NaCl | 100 | + | 71 | + | + | + | 76 | + | - |
| Growth in 15% NaCl | 80 | - | 24 | - | + | + | 29 | - | - |
| Citrate | 0 | - | 12 | - | + | + | 0 | - | - |
| Hydrogen sulphide | 0 | - | 18 | - | - | - | 0 | - | - |
| Motility | 100 | + | 100 | + | + | + | 100 | + | + |
| Starch | 0 | - | 100 | + | + | + | 71 | + | + |
| Glycerol | 100 | + | 41 | + | - | + | 35 | + | + |
| L-arabinose | 40 | + | 53 | + | - | + | 35 | + | - |
| D-ribose | 80 | + | 88 | + | + | + | 65 | + | - |
| D-xylose | 40 | + | 41 | + | - | + | 12 | + | - |
| D-galactose | 20 | + | 12 | - | - | + | 6 | - | - |
| D-glucose | 80 | - | 82 | + | - | + | 82 | + | - |
| D-Fructose | 80 | - | 59 | + | + | + | 82 | + | + |
| D-mannose | 100 | - | 53 | + | - | + | 41 | + | + |
| D-sorbose | 20 | - | 0 | - | - | - | 6 | - | - |
| L-rhamnose | 20 | - | 0 | - | - | + | 6 | - | - |
| Inositol | 20 | + | 18 | + | - | + | 29 | + | + |
| D-mannitol | 80 | + | 53 | + | + | + | 47 | + | + |
| D-sorbitol | 40 | + | 24 | + | + | + | 35 | + | + |

* number of isolates; + positive, - negative

**Continued.**

| Biochemical characteristic | Percentage of positive isolates | | | | | | | | |
| --- | --- | --- | --- | --- | --- | --- | --- | --- | --- |
|  | *B. pumilus* (n=5)* | *B. pumilus* ATCC 7061 | *B. subtilis* group (n=17) | *B. subtilis* ATCC 6633 | *B. amyloliquefaciens* ATCC 23842 | *B. licheniformis* ATCC 14580 | *B. cereus* group (n=17) | *B. cereus* ATCC 6633 | *B. sphaericus* (n=1) |
| Methyl-αD-Mannopyranoside | 20 | - | 18 | - | - | - | 12 | - | - |
| Methyl-αD-Glucopyranoside | 20 | + | 29 | + | - | + | 29 | + | - |
| N-Acylglucosamine | 80 | + | 41 | - | - | + | 82 | - | + |
| Amygdalin | 80 | + | 59 | - | - | + | 41 | - | - |
| Arbutin | 60 | + | 41 | - | - | + | 41 | - | - |
| Esculin ferric citrate | 100 | + | 100 | + | - | + | 100 | + | + |
| Salicin | 60 | + | 76 | + | - | + | 41 | + | - |
| D-cellobiose | 100 | + | 59 | + | + | + | 53 | + | + |
| D-maltose | 40 | + | 65 | + | + | + | 47 | + | - |
| D-lactose | 60 | - | 47 | - | - | - | 18 | + | + |
| D-melibiose | 20 | - | 6 | + | - | + | 12 | - | - |
| D-saccharose | 80 | - | 71 | + | - | + | 59 | + | + |
| D-trehalose | 80 | - | 59 | + | - | + | 71 | + | + |
| Inulin | 0 | + | 6 | + | - | - | 0 | - | - |
| D-raffinose | 20 | + | 18 | + | - | + | 0 | + | - |
| Amidon | 0 | + | 24 | + | - | + | 41 | + | - |
| Glycogen | 0 | + | 12 | + | _ | + | 41 | + | + |
| Gentiobiose | 40 | + | 0 | - | - | - | 12 | + | - |
| D-turanose | 0 | - | 18 | + | - | + | 0 | - | - |
| L-fucose | 20 | - | 0 | - | - | - | 6 | - | - |
| Potassium gluconate | 20 | - | 0 | - | - | - | 0 | - | - |

* number of isolates; + positive, - negative
